# Therapy‐refractory schizophrenia in a patient who previously suffered from Meige’s syndrome

**DOI:** 10.1002/npr2.12081

**Published:** 2019-11-19

**Authors:** Tomihiko Uemura, Masahiko Mochida, Tatsunaru Matsumi, Katsuhiko Yoshimoto, Yoshitaka Tatebayashi

**Affiliations:** ^1^ Tama Hospital Hachioji Japan; ^2^ Affective Disorders Research Project Tokyo Metropolitan Institute of Medical Science Setagaya‐ku Japan; ^3^ Third Department of Internal Medicine Kyorin University School of Medicine Mitaka Japan

**Keywords:** auditory hallucination, basal ganglia, Meige's syndrome, Paliperidone, schizophrenia

## Abstract

The main symptoms of Meige's syndrome are involuntary eye blinking with muddled speech and uncontrollable contraction of the platysma muscle characterized by segmental, primarily oromandibular, dystonia (hyperkinesia). It can also develop after long‐term medication of first‐ and second‐generation antipsychotics. Here, we report the case of a Japanese female schizophrenic patient comorbid with Meige's syndrome and hyperthyroidism. We discuss the relationship between the three diseases, that is, schizophrenia, Meige's syndrome, and hyperthyroidism. Our intention is to consider the important role of the cerebral basal ganglia, where little attention has been given in regard to schizophrenia and Meige's syndrome. A part of this article was presented in a poster section at the joint congress of the 28th Annual Meeting of the Japanese Society of Clinical Neuropsychopharmacology and the 48th Annual Meeting of the Japanese Society of Neuropsychopharmacology held in 2018.

## Abstract 2

We report the case of a Japanese female schizophrenic patient comorbid with Meige's syndrome and hyperthyroidism. We discuss the relationship between the three diseases considering the importance of the basal ganglia, where little attention has been given in regard to schizophrenia and Meige's syndrome.

## Graphical abstract

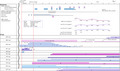

**Abbreviations**
ANP: Asenapine
BNS: Blonanserin
BPD: Biperiden
CPZ: Chlorpromazine
CZP: Clonazepam
DA: Dopamine
HPD: Haloperidol
KYNA: Kynurenic Acid
LZM: Lorazepam
MMI: 1‐Methyl‐2‐mercaptoimidazole (Thiamazole)
MZP: Mirtazapine
OLZ: Olanzapine
PAL: Paliperidone
QTP: Quetiapine
RIS: Risperidone
TSP: Tandospirone
VTA: Ventral tegmental area

## 1 INTRODUCTION

Meige's syndrome is a segmental dystonia, which shows symmetrical blepharospasm and oromandibular cranial dystonia. General dystonia, including segmental dystonia, shows, in contrast to Parkinson's disease, an enhancement of the direct pathway and a decrease in the indirect pathway (Figure 2B)^1^. Additionally, loss of nigral nerve cells and a decrease in nigral melanin deposition have been reported^1^, which suggest the disturbance of dopamine metabolism^1^. Considering the possibility of an abnormal nonmotor circuit in the basal ganglia, the relationship between schizophrenic symptoms (especially an auditory verbal hallucination), Meige's syndrome and hyperthyroidism presented in the case are discussed.

## 2 CASE PRESENTATION

We explained to the patient the details of the drug therapy and described the results and obtained her agreement for publication. A part of her life history was altered to maintain anonymity.

### 2.1 Life history and present anamnesis

She is an only child. Her parents have already passed away. This 55‐year‐old female was suffering for 34 years from schizophrenia diagnosed by the ICD‐10 Classification of Mental and Behavioral Disorders as hebephrenic (disorganized) schizophrenia. Simultaneously, she was diagnosed with Meige's syndrome based on the presence of some blepharospasm symptoms, such as an increased rate of eye blinking and light sensitivity, difficulty keeping her eyes wide open, and oromandibular symptoms, such as trismus. The onset of Meige's syndrome might have been at the age of 33. When she was studying mathematics at a university at 21 years of age, she developed a characteristic silly smile. After graduation, she worked for 2 years at a company but resigned from her job due to delusions of reference and of injury.

She took lessons in various arts such as flower arrangement and tea ceremony in the home. In year X‐14 (33 years of age), she began to dislike her father and had a medical examination in our hospital. In year X‐1 (46 years of age), delusion of injury for a neighbor worsened, and in year X, she was admitted to our hospital. The case report was presented in 2011^2^.

### 2.2 Psychiatric symptoms and drug therapy

#### 2.2.1 The first hospitalization

Following administration of QTP, CZP, and TSP, schizophrenic symptoms such as delusion of reference and that of injury as well as Meige's syndrome symptoms improved at the time, but the auditory hallucinations remained. In year *X* + 10 (56 years of age), she was released from the hospital to a public health care institution and had received regular outpatient treatment every 2 weeks in our hospital. The major antipsychotics were BNS, QTP, ANP, OLZ, HPD, and RIS with LZM and BPD as adjunctive drugs. After approximately 3.5 months, she attempted to automutilate her wrist and was admitted for the second time.

#### 2.2.2 The second hospitalization

**Figure 1:**
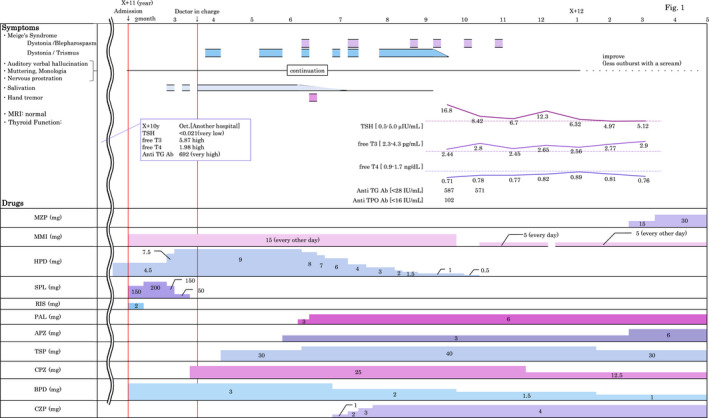
Clinical symptoms and drug therapy

She was treated mainly with ANP and HPD. During her hospitalization, she suffered from irregular bleeding from the genital organs and transferred to a gynecologic hospital. After uterus extirpation following diagnosis of uterocervical cancer, she was admitted for the third time to our hospital. During this hospitalization, her hyperthyroidism was diagnosed as Basedow's disease or Hashimoto's disease (Figure 1).

#### 2.2.3 The third hospitalization

After approximately 2 months in a closed ward, she was transferred to an open ward, and t.he first author worked as a doctor in charge (Figure 1). According to the report of Yoshimura et al^3^, who showed much improvement of Meige's syndrome by changing RIS to PAL, we had chosen PAL as a major antipsychotic with coadministration of CZP to enhance the function of GABAergic neurons and TSP, a 5‐HT1A agonist and known as an antianxiety drug, to improve cognition and improve extrapyramidal disturbance^4^. Schizophrenic symptoms, Meige's syndrome symptoms, analysis of hyperthyroidism, and drug therapy are summarized in Figure 1. Meige's syndrome symptoms (dystonia) improved, but the auditory hallucination speaks in a low voice like a whisper and general fatigue persisted. Thyroid function recovered almost, but not completely, to normal after 7 months (Figure 1).

**Figure 2:**
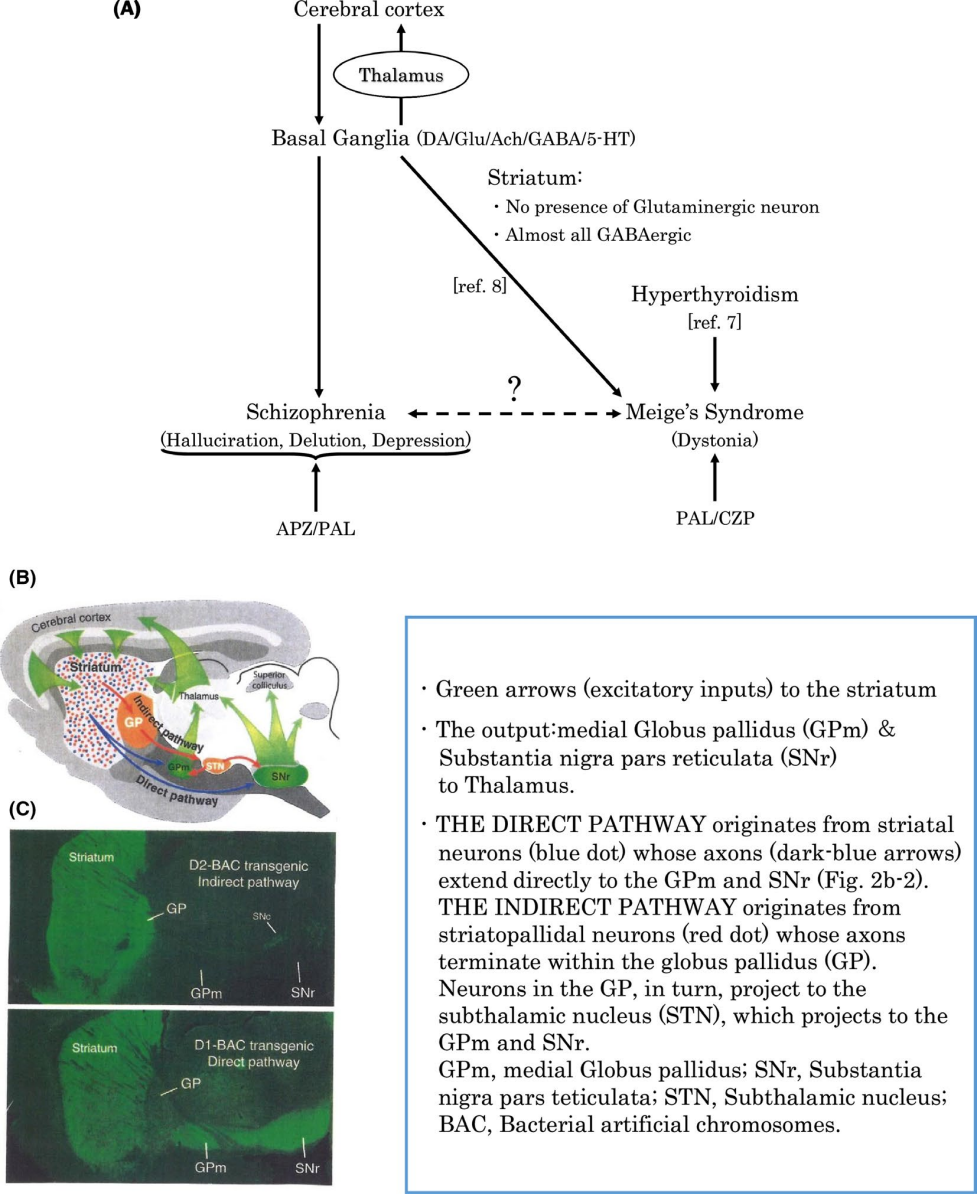
(A) Probable implication of the basal ganglia and thyroid function in three diseases. (B) Circuitry involved in Parkinson's disease^15^ and in general dystonia^1^. (C) D1 and D2 dopamine neurons are segregated to direct and indirect pathway neurons, respectively^15^. Use of the original figure (panels b and c) was permitted by Dr Charles R Gerfen (NIH/NIMH)

## 3 DISCUSSION

The disturbances in the motor circuit in the basal ganglia are associated with Parkinson's disease but often accompanied by psychiatric disorders such as depression and hallucination. On the other hand, the disturbances in the nonmotor circuits are also related to mental illness^5^. We discuss three points below.

1. We begin by considering the importance of the basal ganglia to which little attention has been given regarding nonmotor circuits. Do the three diseases, that is, schizophrenia, Meige's syndrome, and hyperthyroidism, aggregate or are they independent morbidities? A connection of Meige's syndrome with hyperthyroidism^6^ and one of focal dystonia with the basal ganglia^7^ have been reported. We discuss the possible relationship between these three diseases. We examined pathological changes in the basal ganglia by MRI, but no abnormal changes were observed (Figure 1).

After the near normalization of thyroid function, the auditory hallucinations of outbursts with a scream had diminished, but the murmur persisted. What is the biochemical mechanism of a sudden outburst, particularly in a loud voice? Endogenous KYNA (an endogenous metabolite of tryptophan) plays a tonic modulatory role in midbrain DA firing, in which lowered levels of KYNA dampen the activity of midbrain DA neurons^8^. Therefore, endogenous KYNA may reduce the GABAergic neuronal activity projecting to the VTA and disinhibit the VTA DA neurons resulting in an outburst.

Because of the long history of her illness, dopamine supersensitivity psychosis (DSP) cannot be ruled out, but this possibility can be excluded on the diagnostic basis of DSP^9^.

2. Meige's syndrome symptoms were relieved by PAL as reported^3^ and CZP but the auditory hallucinations, monologues, muttering, and nervous prostration had not comparably recovered (Figure 1). She said that her monologues spontaneously came from her mouth repeating what she was hearing. It seemed to be echolalia. This is a symptom of an autopsychic depersonalization, that is, weakness of the self.

We used both PAL and CZP for the treatment of Meige's syndrome, so we cannot compare the mechanisms of the improvement by PAL alone (ie, compared to RIS)^3^. However, the following probable mechanisms of PAL may have contributed: a) its physicochemical property (ie, osmotic‐controlled release oral delivery system), b) its hydroxyl group at position 9 on the tetrahydropyridopyrimidine ring may result in a differential potency of the availability of a second messenger (eg, cAMP, IP3, and Ca^2+^)^10^ between PAL and RIS, and (c) different degrees of 5‐HT7 receptor‐induced inactivation of adenylate cyclase (activated by forskolin) by RIS and PAL^11^, although the participation of 5‐HT receptor subtypes, especially 5‐HT7 receptor in Meige's syndrome, is currently unknown.

3. Regarding the mechanisms of the auditory hallucinations, the self‐monitoring hypothesis^12^ and retrogression toward the bicameral mind^13^ have been proposed. Thought echo (her persistent monologue, see Figure 1) and bicameral mind resemble each other^13^. Auditory hallucinations are an abnormal disposal of the information that comes from self‐speech (inner speech). The origin of an abnormal disposal is laterality^14^ and a bicameral mind^13^. The left brain belongs to oneself (self‐brain), and the right brain belongs to another person (others‐brain). In the future, laterality must be examined to distinguish self and nonself for detailed knowledge of auditory hallucinations.

## CONFLICT OF INTEREST

No potential COI to disclose.

## Data Availability

All the data that are available are shown in the text and Figure 2.
